# The correlation between serum complement levels and clinical presentation in Egyptian immune thrombocytopenia patients

**DOI:** 10.1007/s44313-025-00078-2

**Published:** 2025-05-06

**Authors:** Nourhan Mohamed Nasr, Alia Abdelaziz Ayad, Noha Khalifa Abdelghaffar, Marwa Salah Mohamed

**Affiliations:** 1 Department of Internal Medicine, Faculty of Medicine, Fayoum University, Fayoum, Egypt; 2 Department of Internal Medicine, Faculty of Medicine, Cairo University, Cairo, Egypt; 3 Department of Clinical Pathology, Faculty of Medicine, Fayoum University, Fayoum, Egypt

**Keywords:** Immune thrombocytopenia (ITP), Complement levels, Platelet count

## Abstract

**Background:**

Immune thrombocytopenia (ITP) is an autoimmune condition characterized by low platelet count and increased risk of bleeding. Several pathophysiological processes contribute to the disease, including complement activation by autoantibodies bound to platelet surfaces. This study aimed to assess complement levels in ITP patients and determine their correlation with clinical presentation and disease severity.

**Patients and methods:**

This case–control study enrolled 40 patients (both sexes, aged 18–40 years) with primary ITP and 40 healthy controls. All participants underwent a comprehensive health assessment, thorough physical examination, laboratory investigations, and abdominal ultrasound. These included a complete blood count (CBC) with blood film, renal and hepatic function tests, hepatitis B surface antigen (HBsAg), hepatitis C virus antibodies (HCV-Abs), human immunodeficiency virus (HIV) antibodies, hepatitis B core antibody (HBcAb), C-reactive protein (CRP), antinuclear antibody (ANA), thyroid-stimulating hormone (TSH), erythrocyte sedimentation rate (ESR), serum complement levels (C3 and C4), and *Helicobacter pylori* antigen in stool.

**Results:**

Mean C3 and C4 levels were significantly lower in patients with ITP than in healthy controls. A statistical significant negative correlation was found between CRP and C4 levels in ITP patients. However, no statistically significant relationship was observed between C3 and C4 levels and platelet count in ITP patients, regardless of the presence of bleeding complications.

**Conclusion:**

Complement levels were significantly lower in patients with ITP than in healthy controls. Complement levels were also significantly lower in treatment-naïve patients than in patients who received treatment. Therefore, complement levels could serve as a valuable laboratory test for disease activity.

## Introduction

Immune thrombocytopenic purpura (ITP) is an autoimmune disorder characterized by thrombocytopenia and hemorrhagic events resulting from antiplatelet autoantibodies. ITP is typically classified into two categories: primary and secondary. Primary ITP is an acquired ITP, whereas secondary ITP is thrombocytopenia induced by an alternative illness. These disorders include various autoimmune diseases (such as systemic lupus erythematosus and rheumatoid arthritis), chronic infections (including hepatitis C, human immunodeficiency virus [HIV], and *Helicobacter pylori*), lymphoproliferative neoplasms (such as chronic lymphocytic leukemia and lymphoma), and a range of medications and vaccines [1]. A significant feature of ITP is the variation among patients in both clinical profiles and therapeutic responses, with a minority classified as refractory. This heterogeneity is likely attributable to the involvement of multiple pathophysiological pathways that affect individual cases to varying degrees [2].

Enhanced platelet destruction and reduced platelet synthesis due to immunological intolerance have been implicated in the etiology of ITP [3]. Antibody-mediated cytotoxicity is facilitated by several mechanisms, including complement-dependent cytotoxicity (CDC), which leads to the destruction of target cells through the formation of a membrane attack complex, and antibody-dependent cellular phagocytosis (ADCP), which promotes phagocytosis by macrophages that express Fc gamma receptors (FcR) and complement receptor 1, which bind to the complement component C3b [4]. In ITP, the role of antibody-dependent cellular cytotoxicity (ADCC) initiated by natural killer cells is likely to be diminished [5].

The complement system is believed to play a role in the pathophysiology of ITP in certain individuals. However, the precise role of complement in ITP remains unclear. Preliminary investigations involving sutimlimab, a monoclonal IgGC1 s inhibitor, have been conducted in a study that included adult patients with ITP for over one year and an insufficient response to more than two prior therapies. Twelve patients were treated, with 42% exhibiting a response (platelet count > 50 × 10^9/L). Four patients (33%) achieved platelet counts exceeding 50 × 10^9/L during more than 70% of their study visits. One-third of the patients exhibited a response within two days or less. No therapy-related adverse effects were observed [6]. Therefore, complement levels appear to play a role in the pathophysiology of ITP and may influence its clinical presentation and treatment response. Consequently, this study evaluated the complement levels in ITP patients.

### Patients and methods

This case–control study included adult ITP patients. The study adhered to the Institutional Committee Regulations for the Protection of Human Subjects adopted by the 18 th World Medical Assembly in Helsinki, Finland. The study protocol was submitted for approval by the Ethics Committee of Cairo University (*Clinical trial number: not applicable.*).

This research comprised two groups:

- *Group 1* comprised 40 patients (both sexes, aged 18–45 years) with primary ITP (diagnosed according to ASH 2019 clinical practice guidelines) [7]. The patients were divided into two subgroups: 20 treatment-naïve patients and 20 patients who had received treatment. All patients presented to the hematology clinics at Al Kasr Alainy for diagnosis or medical follow-up during the study period.

Patients were classified into two groups based on bleeding complications: those with and without bleeding complications. Bleeding complications were defined as the presence of hemorrhage requiring therapy at presentation, or the development of new bleeding symptoms necessitating additional treatment with an alternative platelet-enhancing drug or a higher dose [8].

The exclusion criteria included complete remission of ITP, rheumatologic illnesses (such as systemic lupus erythematosus) or any other clinical conditions recognized as complement-mediated or leading to diminished serum complement concentrations, concurrent hemolytic anemia (Evans syndrome), familial predisposition to platelet or coagulation disorders, and pregnancy.

- *Group 2*: The control group comprised 40 healthy volunteers matched for age and sex with the case group.

Informed consent was obtained from all participants prior to their participation in the study. All patients underwent comprehensive medical history assessment, thorough physical examination, abdominal ultrasonography, and laboratory testing, including complete blood count (CBC) and blood film, renal and hepatic function tests, hepatitis C virus antibodies (HCV-Abs), hepatitis B surface antigen (HBsAg), hepatitis B core antibody (HBcAb), HIV antibodies, antinuclear antibody (ANA), C-reactive protein (CRP), erythrocyte sedimentation rate (ESR), thyroid-stimulating hormone, and Helicobacter pylori antigen in stool. Serum complement levels of C3 and C4 were measured using an immunoturbidimetric test on a Cobas C analyzer (Roche Diagnostics GmbH, Sandhofer Strasse 116, D- 68305 Mannheim). Data on complement test results (including illness status and platelet count at the time of testing), patient information, and illness characteristics (medication history and therapy at the time of complement testing) were collected for analysis.

### Statistical analysis

The data were gathered, encoded for manipulation, and double-entered into Microsoft Access software. Analyses were conducted using SPSS software version 18 on Windows 7. Descriptive analyses included numerical values and percentages for qualitative data, and arithmetic means (measures of central tendency) and standard deviations (measures of dispersion) for quantitative parametric data. Inferential statistical tests were employed as follows: One-way ANOVA for comparing multiple independent groups of quantitative parametric data, chi-square test for comparing two or more qualitative groups, and bivariate correlation tests for assessing correlations among variables. Statistical significance was determined using a *P*-value threshold of less than 0.05.

## Results

### Case information & baseline characteristics

Case information and baseline characteristics are presented in Table 1.

**Table 1:** Case information and baseline characteristics

	ITP Cases	Control	*P* value
**Parameter**	**Mean ± SD**	**Mean ± SD**	**0.705**
**Age**	34.18 ± 11.27	33.25 ± 10.53	
Sex	ITP Cases (%)		Control (%)
Male	5 (12.5%)		6 (15%)
Female	35 (87.50%)		34 (85%)
**Treatment**	**Count (%)**		
No treatment	20 (50.00%)		
Corticosteroid	11 (27.50%)		
Steroid + azathioprine	3 (7.50)		
Eltrombopag	1 (2.50%)		
Steroid + splenectomy	2 (5.00%)		
Ritxumab	3 (7.50%		

ITP patient ages ranged from 18 to 45 years, with a mean age of 34.18 ± 11.2 years. The cohort comprised 87.5% females and 12.5% males. Twenty patients were treatment-naïve, eleven were on corticosteroids, three were on corticosteroids and immunosuppression (azathioprine), three were on rituximab, two had undergone splenectomy, and one was on eltrombopag.

The laboratory parameters are listed in Table 2. The mean hemoglobin level was statistically significantly different between cases and controls. The mean hemoglobin level of ITP patients was 11.33 ± 1.81 g/dl, while the mean level of the control group was 13.38 ± 1.57 g/dl. The mean platelet count was also statistically significantly different between the cases and controls. The mean platelet count of ITP patients was 32.1 ± 29.6 × 10^9^/L, while the mean level of the control group was 228.25 ± 59.65 × 10^9^/L.

**Table 2:** Laboratory investigations of the study groups at the beginning of the study

	**ITP cases**		**Control**		***P* value**
	**Mean**	**Standard Deviation**	**Mean**	**Standard Deviation**	
**HB** **(gm/dl)**	11.33	1.81	13.38	1.57	< 0.001 *
**PLT** **(10**^**3**^**/UL)**	32.10	29.65	228.25	59.65	< 0.001 *
**TLC** **(10**^**3**^**/UL)**	7.77	3.71	7.02	3.18	0.416
**MPV** **fl**	9.68	1.32	9.51	. 1.14	0.528
**S Urea** **mg/dl**	27.60	8.62	27.68	8.89	0.970
**Creatinine** **mg/dl**	0.73	0.19	0.72	0.19	0.953
**ALT** **(U/L)**	23.45	18.03	19.88	7.23	0.764
**AST** **(U/L)**	19.55	9.06	20.10	10.70	0.873
**CRP** **mg/dL**	8.16	4.33	3.30	1.14	< 0.001 *
**ESR** **mm/h**	21.15	13.37	19.37	13.53	0.327
**TSH** **mIU/L**	1.47	0.84	1.60	0.99	0.689
**C3** **mg/dL**	125.10	28.15	143.80	23.29	0.002
**C4** **mg/dL**	22.72	10.33	30.53	5.66	< 0.001 *

^*^Statistically significant at *p* < 0.05

**Fig. 1:**
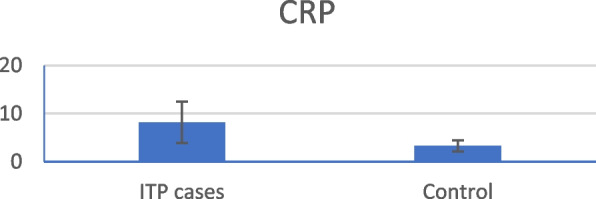
Mean CRP level in ITP patients versus control group

**Fig. 2:**
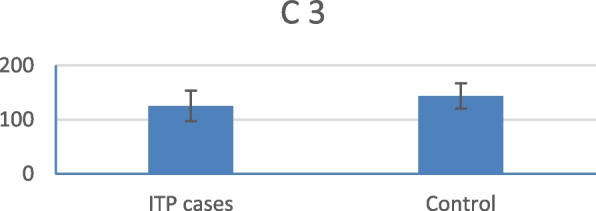
Mean C3 level in ITP patients versus control group

**Fig. 3:**
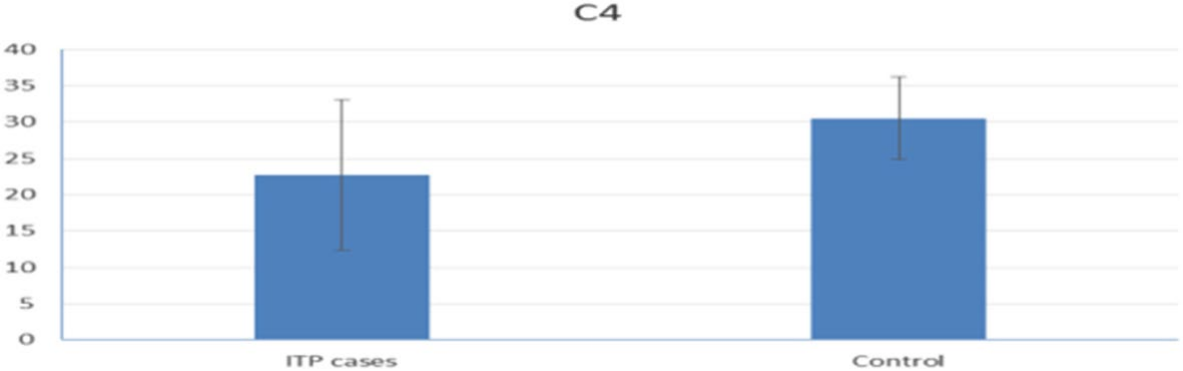
Mean C4 level in ITP patients versus control group

CRP levels were statistically significantly higher in ITP patients than in the control group (Fig. 1). Conversely, kidney function tests, liver function tests, and ESR were not statistically significantly different between the groups. The mean C3 and C4 levels were significantly lower in patients with ITP than in healthy subjects (Figs. 2 and 3).

### Complement activity in ITP: Treatment-Naïve versus Treated Patients

**Fig. 4:**
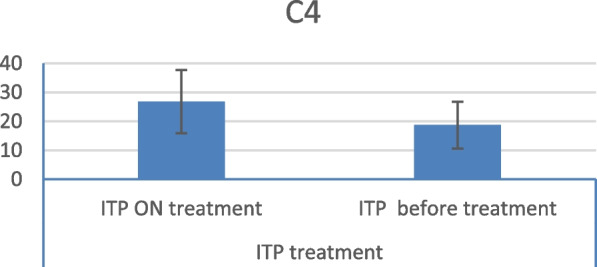
Mean C4 level in ITP patients on treatment and naive patients

A comparison of complement assay results between treatment-naïve patients and patients with ITP receiving treatment revealed a statistically significant decrease in the mean C4 level in the treatment-naïve group (Fig. 4).

### Correlation between plasma level of C3 and C4, platelet count, MPV, ESR, and CRP

**Fig. 5:**
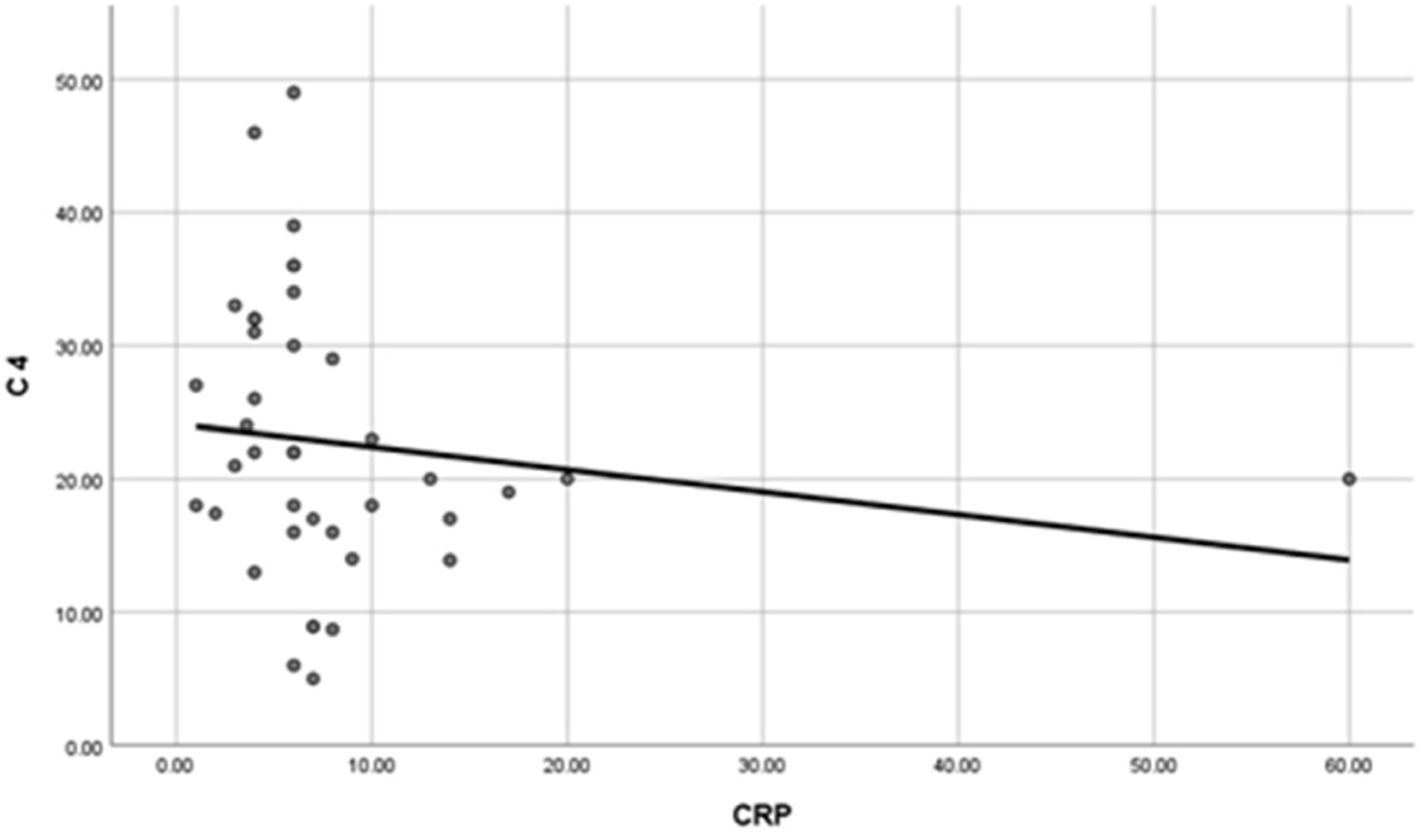
Correlation between plasma levels of C4 and CRP

The correlations between platelet count and serum C3 and C4 levels in patients were not statistically significant. Similarly, no statistically significant relationship was found between mean platelet volume and complement levels (Table 3). While correlations between ESR and serum C3 or C4 levels were also not statistically significant in ITP patients, a statistically significant negative correlation was observed between CRP and C4 levels (Table 3, Fig. 5).

**Table 3:** Correlations between plasma levels of C3 and C4 and platelet count, MPV, ESR, CRP

		**C 3**	**C 4**
**PLT**	**Correlation Coefficient**	0.004	0.173
	**P value**	0.979	0.285
	**N**	40	40
**MPV**	**Correlation Coefficient**	0.050	− 0.043-
	**P value**	0.761	0.794
	**N**	40	40
**CRP**	**Correlation Coefficient**	− 0.278-	− 0.383-
	**P value**	0.082	0.015 *
	**N**	40	40
**ESR**	**Correlation Coefficient**	− 0.159-	− 0.138-
	**P value**	0.327	0.395
	**N**	40	40

^*^Statistically significant at *p* < 0.05

### Bleeding complications

Patients with bleeding complications had significantly lower platelet counts than those without complications. The mean platelet count in patients with bleeding complications was 15.90 ± 15.25 × 10^9^/L, compared with that of 51.89 ± 31.2 × 10^9^/L in patients without bleeding complications. No significant difference was observed in complement levels between patients with and without bleeding complications. Correlation analysis between C3, C4, and platelet count in ITP patients with and without bleeding complications, revealed no statistically significant relationship (Table 4).

**Table 4:** Comparison of platelet count and C3 and C4 levels between ITP patients with and without bleeding complications

					***P* value**
	**patients with bleeding complications**		**patients without bleeding complications**		
	**Mean**	**Standard Deviation**	**Mean**	**Standard Deviation**	
**PLT(10**^**3**^**/UL)**	15.90	15.25	51.89	31.23	< 0.001 *
**C 3 mg/dL**	125.83	24.72	124.50	31.24	0.476
**C 4 mg/dL**	24.52	11.35	21.25	9.42	0.492

^*^Statistically significant at *p *< 0.05

## Discussion

Primary ITP is an acquired autoimmune disorder characterized by a persistently low isolated platelet count (< 100 × 10^9/L), resulting from a disruption of immunological homeostasis, which favors autoimmunity [9]. Despite extensive research on ITP pathophysiology in recent years, our understanding remains incomplete [10]. A notable feature of ITP is the variability in clinical profiles and therapeutic responses among patients, with a small percentage classified as refractory [2]. This heterogeneity likely stems from the involvement of diverse pathophysiological mechanisms affecting individuals to varying degrees. These mechanisms include complement activation by autoantibodies bound to platelet surfaces. Consequently, this study aimed to evaluate complement levels in ITP patients and determine the correlation between these levels, clinical presentation, and disease severity.

The study revealed that CRP levels were significantly elevated in ITP patients compared with those in the control group. These findings are consistent with those of a study by Pan et al., who suggested that CRP may act as a novel pathogenic cofactor, enhancing IgG-mediated phagocytic responses, and leading to thrombocytopenia. Elevated CRP levels at diagnosis predicted lower platelet counts and increased clinical bleeding severity in patients with ITP [11]. Conversely, Dal et al. reported no significant difference (*p* > 0.05) in CRP levels between ITP patients and the control group. [12].

In this study, the mean C3 and C4 levels in ITP patients were within the normal reference ranges. However, they were significantly lower in ITP patients than in healthy controls. Furthermore, a comparison between treated and treatment-naïve patients showed a statistically significant decrease in the mean C4 levels in the treatment-naïve group.

These findings align with those of Cheloff et al., who observed diminished complement levels in one-third of ITP patients, which correlated with increased disease severity [13]. Conversely, another study found no statistically significant differences in plasma C3 or C4 levels between ITP patients and controls [14].

The observation of a statistically significant decrease in mean C4 levels in treatment-naïve patients compared with levels in those receiving treatment supports the findings of Cheloff et al., who reported that complement may significantly contribute to platelet loss in a subset of ITP patients. Moreover, they reported that patients requiring therapy exhibited markedly reduced C4 and CH50 levels compared with those who did not [13]. Interestingly, no significant variations in complement levels were observed between patients with and without identifiable platelet autoantibodies, which is counterintuitive, given that complement fixation by platelet autoantibodies is a key mechanism of complement-mediated platelet destruction [15, 16].

Assessment of correlations between ESR and serum C3 and C4 levels revealed no statistically significant relationship in ITP patients. However, a statistically significant negative correlation was found between the CRP and C4 levels. CRP enhances antibody-mediated platelet destruction by acting as a ligand for phagocyte FcγR and FcαRI receptors, both of which can bind CRP, hence facilitating phagocytic destruction. This suggests that addressing the underlying inflammation in addition to treating thrombocytopenia may be beneficial for ITP patients [17].

No significant differences in complement levels were observed between patients with and without bleeding complications. Similarly, no statistically significant relationship was found between C3 and C4 levels and platelet counts in ITP patients, regardless of bleeding complications. These results are consistent with those of Castelli et al., who reported no variation in the sC5b- 9 concentration between patients with and without blood loss complications [14].

All patients with bleeding complications had significantly lower platelet counts. The mean platelet count in patients with high-risk bleeding was 15.90 ± 15.25 × 10^9^/L, while the mean platelet count in patients with low-risk bleeding was 51.89 ± 31.2 × 10^9^/L. These findings align with those of Piel-Julian et al., who reported that bleeding occurred more frequently in patients with a platelet count below 20 × 10^9/L, with an incidence of 86% (135/157) in this group compared with 27.6% (40/145) in patients with a platelet count of 20 × 10^9/L or greater. Hemorrhage was observed in 94.8% (110/116) of patients with a platelet count below 10 × 10^9/L. Mucosal hemorrhage occurred more frequently in participants with platelet counts below 20 × 10^9/L (45.2%, 71/157) than in those with platelet counts above 20 × 10^9/L (13.8%, 20/145) [18].

Interestingly, this study suggests that complement levels could be valuable for clinical monitoring of disease activity. A larger prospective multicenter study comparing the complement levels before and after treatment is recommended to confirm our findings.

## Conclusion

Complement is thought to play a role in the pathophysiology of ITP in certain individuals. Complement levels were significantly lower in treatment-naive patients than in healthy participants. Furthermore, complement levels were significantly lower in treatment-naive patients than in patients undergoing treatment. Therefore, complement levels could serve as a valuable laboratory test for the assessment of disease activity**.**

## Data Availability

No datasets were generated or analysed during the current study.

## Abbreviations

ADCC: Antibody-dependent cellular cytotoxicity
ADCP: Antibody-dependent cellular phagocytosis
ALT: Alanine transaminase
ANA: Antinuclear antibody
AST: Aspartate aminotransferase
C3: Complement component 3
C4: Complement component 4
CBC: Complete blood count
CDC: Complement-dependent cytotoxicity
CRP: C*-*reactive protein
ESR: Erythrocyte sedimentation rate
FcR: Fc gamma receptors
HB: Hemoglobin
HBcAb: Hepatitis B core antibody
HBsAg: Hepatitis B surface antigen
HCV-Abs: Hepatitis C virus antibodies
HIV: Human immunodeficiency virus
MPV: Mean platelet volume
PLT: Platelet
TLC: Total leucocytic count
TSH: Thyroid stimulating hormone
